# Yueju Pill Rapidly Induces Antidepressant-Like Effects and Acutely Enhances BDNF Expression in Mouse Brain

**DOI:** 10.1155/2013/184367

**Published:** 2013-04-22

**Authors:** Wenda Xue, Xin Zhou, Nan Yi, Lihua Jiang, Weiwei Tao, Runjie Wu, Dan Wang, Jingjing Jiang, Xiaoyin Ge, Yuyue Wang, Haoxin Wu, Gang Chen

**Affiliations:** ^1^Center for Translational Systems Biology and Neuroscience, School of Basic Biomedical Science, Nanjing University of Chinese Medicine, Nanjing 210023, China; ^2^Laboratory of Integrative Biomedicine of Brain Diseases, School of Basic Biomedical Science, Nanjing University of Chinese Medicine, Nanjing 210023, China; ^3^TCM Jingui Research Section, School of Basic Biomedical Science, Nanjing University of Chinese Medicine, Nanjing 210023, China; ^4^School of Life Science, Nanjing Normal University, Nanjing 210023, China; ^5^Medical School, Jinan University, Guangzhou 510632, China

## Abstract

The traditional antidepressants have a major disadvantage in delayed onset of efficacy, and the emerging fast-acting antidepressant ketamine has adverse behavioral and neurotoxic effects. Yueju pill, an herb medicine formulated eight hundred years ago by Doctor Zhu Danxi, has been popularly prescribed in China for alleviation of depression-like symptoms. Although several clinical outcome studies reported the relative short onset of antidepressant effects of Yueju, this has not been scientifically investigated. We, therefore, examined the rapid antidepressant effect of Yueju in mice and tested the underlying molecular mechanisms. We found that acute administration of ethanol extract of Yueju rapidly attenuated depressive-like symptoms in learned helpless paradigm, and the antidepressant-like effects were sustained for at least 24 hours in tail suspension test in ICR mice. Additionally, Yueju, like ketamine, rapidly increased the expression of brain-derived neurotrophic factor (BDNF) in the hippocampus, whereas the BDNF mRNA expression remained unaltered. Yueju rapidly reduced the phosphorylation of eukaryotic elongation factor 2 (eEF2), leading to desuppression of BDNF synthesis. Unlike ketamine, both the BDNF expression and eEF2 phosphorylation were revered at 24 hours after Yueju administration. This study is the first to demonstrate the rapid antidepressant effects of an herb medicine, offering an opportunity to improve therapy of depression.

## 1. Introduction

Major depressive disorder (MDD, or depression) has a lifetime prevalence of about 20% and becomes the second leading cause of disease burden worldwide [1]. MDD is commonly treated with monoamine-based antidepressants such as serotonin selective reuptake inhibitors. However, about 1/3 of depressives who seek the treatment are nonresponsive to conventional antidepressants [2]. Moreover, conventional antidepressants have a major disadvantage in their several-weeks-long lag period of therapeutic efficacy, which causes discontinuation of the medication and is dangerous for those suicide-risk patients [3, 4]. Clearly, it is of great interest to develop faster acting and more effective antidepressants. Recent clinical and animal studies demonstrate that a subanesthetic dose of ketamine can quickly alleviate depressive syndromes, and this effect can last for a few days long [5–7]. Experimental studies also suggest that the rapid enhancement of brain derivative neurotrophic factor (BDNF) expression and synaptic plasticity in given brain regions are responsible for the effect [8, 9]. However, concerns regarding the adverse behavioral and neurotoxic effects and abuse potential of ketamine largely restrict its clinical utility. 

 Traditional Chinese medicine (TCM) has been widely used for the treatment of depression. Several ancient herb formula prescriptions designated to treat depressive-like symptoms are still frequently prescribed alone or adjunctive with conventional antidepressants nowadays in China. Recently, researchers have confirmed the effectiveness of herbal medicine therapies on depression using more scientifically rigorous double-blind, placebo-controlled paradigms [10]. Additionally, a growing number of studies using animal models demonstrated the antidepressant features of herbal medicines and revealed the underlying mechanisms, including the increase in BDNF [11, 12]. However, none of the previous studies tested whether an antidepressant herbal medicine acts quickly or enduringly.

“Yueju” (namely, depression-overcoming) pill, formulated 800 years ago by a famous Chinese medicine Doctor Zhu Danxi, is popularly prescribed to treat depression, anxiety, and irritability. Yueju consists of identical amount of five herbs, Xiang Fu, Chuan Xiong, Zhi Zi, Cang Zu, and Shen Qu. The rationale for Yueju, in terms of TCM, is to liberate Qi from stagnation and eradicate specific syndromes of depression by individual herbs, that is, Qi depression by Xiang Fu, blood depression by Chuan Xiong, fire depression by Zhi Zi, damp depression by Cang Zu, and food depression by Shen Qu. Previous animal studies have shown that Yueju is an effective antidepressant [13, 14]. In fact, a few case study reports indicate a relative short onset of the antidepressant efficacy of Yueju, but this has not been scientifically investigated. Here, using animal models of depression, we tested whether Yueju rapidly and lastingly act to alleviate the depressive symptoms and examined the underlying molecular mechanisms. 

## 2. Materials and Methods

### 2.1. Animals

Male outbred ICR were used in the present study. Mice aged 6–8 weeks old (18–24 g) were habituated to animal facilities for 1 week before behavioral testing. Mice were kept on a 12 h/12 hr light/dark cycle and were given free access to food and water. For all behavioral testing, mice were weight matched. All animal procedures conformed to the Guide for the Care and Use of Laboratory Animals and were approved by the Institutional Animal Care and Use Committee at Nanjing University of Chinese medicine. The experimenters were blinded to the assignments of the mice.

### 2.2. Drugs

The medicinal plants used to prepare Yueju are *Cyperus rotundus *L. (Xiang Fu), *Ligusticum chuanxiong *Hort. (Chuan Xiong), *Gardenia jasminoides *Ellis. (Zhi Zi), *Atractylodes lancea *(Thunb.) DC. (Chang Zu), and *Massa Fermentata *(Shen Qu). All the medicinal plants were purchased from Nanjing Guoyi Clinical, Medicinal Material Department (Nanjing, China) and authenticated by Dr. Yang Lianyun, Department of Chinese Materia Medica, Nanjing University of Chinese Medicine. According to the preparation of Yueju formula, five plant materials weighted 200.0 g were powdered into 100 meshes. The power was immersed with 2 L 95% ethanol for 2 days at room temperature, and the dissolved solution was collected. This procedure was repeated twice. The collected extracts were combined, filtered, and dried under reduced pressure at a temperature below 55°C. The yielding product weighted about 48.4 g. Yueju ethanol extract (YJ-E) was dispersed in Tween 80 solution (0.5%, w/v in saline). Vehicle solvent (0.5%, w/v Tween 80 in saline) served as the negative control. The solutions of the herb preparation and vehicle were administered to the mice via intragastric administration of the solution at a dosage of 0.2 mL/20 g (body weight), and the concentration of the solution was 300 mg/mL. Ketamine HCl (Gutian Pharmaceuticals, China), dissolved in saline, was administered intraperitoneally.

### 2.3. The Quality and Constitutes of Yueju Ethanol Extract

Twenty-three batches of YJ-E were analyzed using HPLC fingerprint analysis. The HPLC analysis was performed on a Waters 2695 Alliance HPLC system (Waters Corp., Milford, MA, USA), equipped with a quaternary pump solvent management system, an on-line degasser, and an autosampler. The raw data were detected with 2998 DAD and processed with Empower Software. An Apollo C_18_ column (250 mm × 4.6 mm, 5 *μ*m) preceded by a Waters Symmetry Shield RP C_18_ guard column (20 mm × 3.9 mm, 5 *μ*m) was applied for all analyses. The injection volume was 10 *μ*L, and the column temperature was maintained at 30°C. The DAD detector was set at 260 nm for acquiring chromatograms. The mobile phase was composed of A (acetonitrile) and B (0.1% aqueous acetate acid, *v*/*v*) with a gradient elution: 0–20 min, 10–20% A; 20–35 min, 20–58% A; 35–55 min, 58–85% A; 55–60 min, 85–100% A; and 60–63 min, 100% A. The flow rate of the mobile phase was 1.0 mL min^−1^.

HPLC-DAD chromatographic data of the 23 tested samples were submitted for analysis by using the professional software “Similarity Evaluation System for Chromatographic Fingerprint of TCM” (Version 2004 A) to extract the mean chromatogram and the similarities.

### 2.4. Learned Helplessness Paradigm

The procedures for learned helplessness were followed as reported [15, 16]. Learned helplessness experiments were performed in soundproofed two-way shuttle boxes (40 × 10 × 13 cm), with walls made of clear Plexiglas. The chamber was divided into two identical compartments. For the induction of helplessness, mice received 120 inescapable shocks (18–44 s, average 30 s; 0.45 mA for 15 s) once daily for 2 consecutive training days. For screening of helpless mice, animals were subjected to 30 avoidance trials (18–44 s, average 30 s; 0.45 mA for 3 s). Mice that developed helplessness (>10 escape failures) were treated with either Yueju or vehicle and were tested for helplessness 24 hours after the treatment. 

### 2.5. Open Field Test

The open field test estimated locomotor activity and anxiety-like behavior in a bright-lighted, open area. Testing was performed for 5 min in a well-illuminated (~300 lux) transparent acrylic cage (40 × 40 × 15 cm). Test compounds and vehicle were administered 30 min prior to testing. The activities of mice in two compartments, a compartment near the walls and a central area compartment, were tracked. Both the distance traveled (cm) and time spent in compartments were analyzed. Testing apparatus was thoroughly cleaned before each animal using 70% ethanol. 

### 2.6. Tail Suspension Test

Mice were assessed in the TST, which was performed with a computerized device allowing four animals to be tested at one time. In a chamber both acoustically and visually isolated, an individual mouse was suspended 50 cm above the floor by adhesive tape placed approximately 1 cm from the tip of the tail. The activities of the animals were videotaped. The computer calculated the total duration of immobility during the last 4 min in a 6-min testing time [17]. Mice were returned in individual cages and remained so until the end of the experiment.

### 2.7. Quantitative Real-Time PCR Analysis

RNA was isolated from the whole hippocampus (ventral and dorsal) using Trizol reagent (Invitrogen) and were reverse transcribed to cDNA using the SYBR PrimeScript RT-PCR Kit (Takara). Quantitative RT-PCR was performed with 1.5 *μ*L of cDNA using the SYBR Green Master Mix reagent (Takara). The primer showed the following: BDNF forward, 5′-CCA TAA AGG ACG CGG ACT TGT ACA-3′; BDNF reverse, 5′-AGA CAT GTT TGC GGC ATC CAG-3′; GAPDH forward, 5′-AAC GAC CCC TTC ATT GAC-3′; and GAPDH reverse, 5′-TCC ACG ACA TAC TCA GCA C-3′. The fold-change in BDNF expression (coding exon) was normalized to GAPDH. The qPCR was carried out based on manufacture's manual. Relative expression values were obtained by the ΔΔCT method.

### 2.8. Western Blot

The whole hippocampus (ventral and dorsal) was lysed in RIPA buffer containing protease inhibitors and phosphatase inhibitors. Protein concentration was determined colorimetrically by BCA assay (Pierce, Rockford, IL, USA). Protein lysates were separated by 12% SDS-PAGE electrophoresis and were transferred onto polyvinylidene difluoride (PVDF) membranes. After blocking with 5% BSA for 1 hr, the membranes were incubated with BDNF (Santa Cruz Biotechnology, sc-546, 1 : 200), p-eEF2 (Cell Signaling Technology, 2331, 1 : 500), eEF2 (Cell Signaling Technology, 2332, 1 : 500), and GAPDH (Invitrogen, AM4300, 1 : 2000) antibodies at 4°C overnight, followed by incubation with horseradish peroxidase-conjugated secondary antibodies for 1 hr. Then, the blots were visualized using the SuperSignal West Pico Chemiluminescent Substrate (Thermo Fisher Scientific Inc.). BDNF and pro-BDNF were normalized to GAPDH bands, and p-eEF2 and total eEF2 bands were taken as a ratio of GAPDH bands. All experiments were performed 3 times. 

### 2.9. Statistics Analyses

Two-sample comparisons were carried out using the two-tailed Student's *t*-test; multiple comparisons were made using one-way ANOVA, followed by the Newman-Keuls multiple range test. All data are presented as Mean ± SEM, and statistical significance was accepted at the 5% level unless otherwise indicated.

## 3. Results

### 3.1. Fingerprint of Ethanol Extract of Yueju

**Figure 1:**
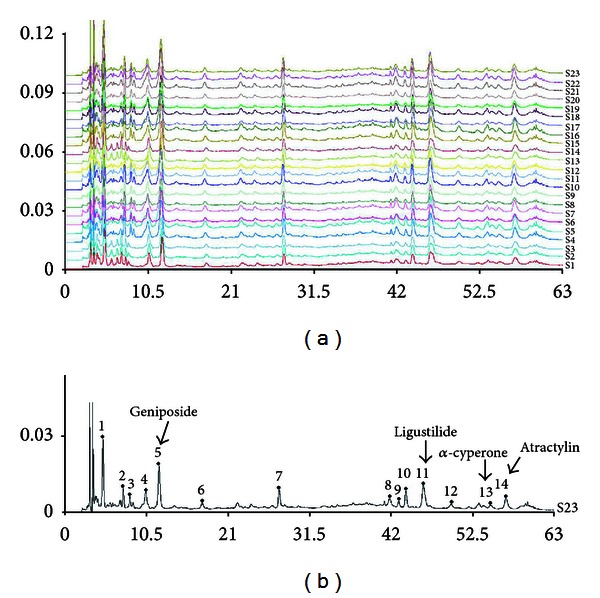
Fingerprints of YJ-E. (a) The chromatographic fingerprints of twenty-three samples of YJ-E (S1–S23) and (b) the mean chromatographic fingerprint developed with the software “Similarity Evaluation System for Chromatographic Fingerprint of TCM.” The 14 common peaks are labeled.

Twenty-three samples were used to develop the standard fingerprints (Figure 1(a)). The mean chromatographic fingerprint obtained from the software “Similarity Evaluation System for Chromatographic Fingerprint of TCM” was shown in Figure 1(b). Peaks presented in all 23 samples were defined as “common peaks.” As a result, 14 characteristic peaks shown in the fingerprint chromatogram were assigned as common peaks. Of these, 4 peaks were identified by comparison of their retention times (RTs) and UV absorption spectra with those of standard compounds (geniposide, ligustilide, *α*-cyperone, and atractylin). The similarity of each chromatogram to the mean chromatogram was greater than 0.9, indicating that the quality of the 23 batches was very similar and suitable.

### 3.2. Acute Yueju Administration Showed a Rapid Antidepressant Effect in Learned Helpless Paradigm

**Figure 2:**
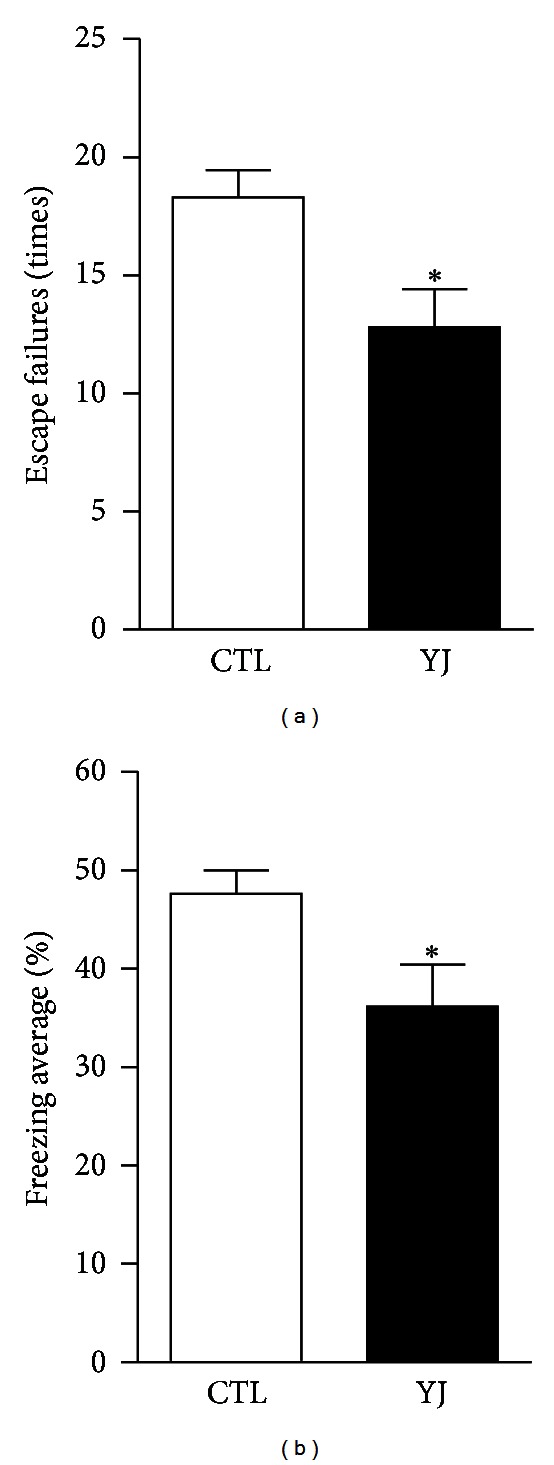
A rapid antidepressant-like behavioral responses by Yueju in the learned helpless paradigm. (a) Learned helplessness was quantified as number of escape failures in an active avoidance test. Mice received inescapable shock training for two days prior to a 30-trial active avoidance test. Escape failures were decreased in mice given Yueju (YJ). (b) The mean percent shock-elicited freezing for the entire observation period was also reduced in mice given YJ. *P < 0.05, two-tailed *t*-test. Data represent mean ± SEM. *n* = 8.

Learned helplessness (LH) paradigm was employed to test the rapid efficacy of Yueju on relieving depressive-like symptoms. In LH paradigm, only after several days of repeated administration of conventional monoaminergic antidepressants, mice start to show response to the treatment [18]. In the mice that developed helplessness, a single administration of Yueju significantly reduced the number of escape failures (Figure 2(a)) (*P* = 0.013, *n* = 8, and two-tailed *t*-test) and the freezing (Figure 2(b)) (*P* = 0.039). The latency to escape also showed a trend to decrease in Yueju-treated mice in this system (*P* = 0.12). These results imply that in mice, the acute administration of Yueju exerts rapid antidepressant-like effects.

### 3.3. Antidepressant-Like Effects of Yueju Sustained for 24 Hours in ICR Mice

**Figure 3:**
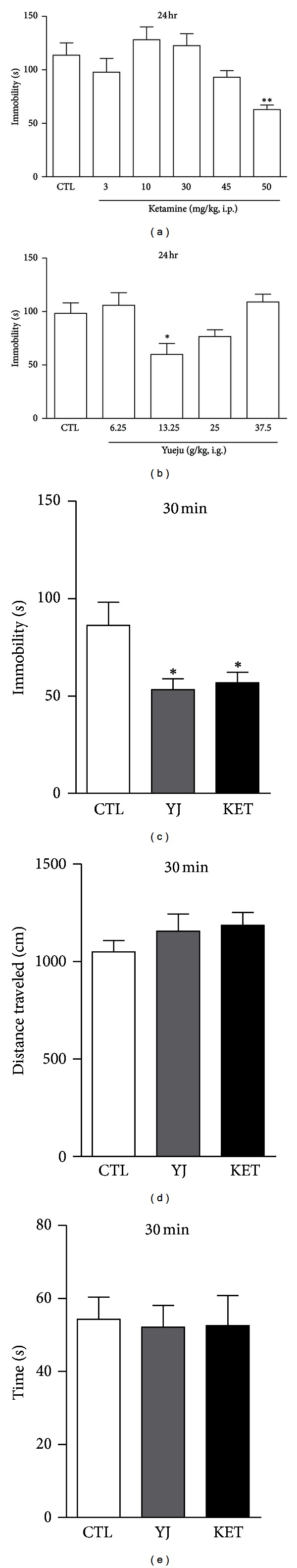
Effects of ketamine and Yueju on the tail suspension test and open field test. (a) Tail suspension test was carried out at 24 hours after ketamine (KET) administration. Immobility time was measured for the last 4 minutes during the 6 minutes testing time. one-way analysis of variance (ANOVA), *F*_(5,51)_ = 7.956, *P* < 0.001, and *n* = 8–11. (b) Tail suspension test at 24 hours after Yueju (YJ) administration. Immobility time was measured for the last 4 minutes during the 6 minutes testing time. ANOVA, *F*_(4,64)_ = 4.611, *P* = 0.003, and *n* = 12–15. (c) Tail suspension test at 30 min after drug administration. ANOVA, *F*_(2,24)_ = 4.875, *P* = 0.017, and *n* = 8–10. Both YJ and ketamine (KET) significantly reduced the immobility time, compared to vehicle. (d) Total distance traveled during a 5 munities open field testing time, ANOVA, *F*_(2,30)_ = 0.9903, *P* = 0.384, and *n* = 11. (e) Time spent on the center part during a 5 munities open field testing time. ANOVA, *F*_(2,30)_ = 0.0276, *P* = 0.973, and *n* = 11. The open field testing started at 30 minutes after drug treatment. *P < 0.05 and **P < 0.01. Data represent mean ± SEM.

To determine how long the antidepressant-like effects of Yueju sustained in mice, TST behavior despair paradigm was used. At 24 hours after administration of different doses of ketamine (Figure 3(a)) or Yueju (Figure 3(b)) in ICR mice, there were significant dose effects for ketamine (ANOVA, *F*_(5,51)_ = 7.956, *P* < 0.001, and *n* = 8–11), and the dose of 50 mg/kg effectively reduced the immobility time (*P* = 0.016). Yueju also showed dose effects at 24 hours after administration (ANOVA, *F*_(4,64)_ = 4.611, *P* = 0.003, and *n* = 12–15). The effective dose was 13.5 g/kg Yueju (*P* = 0.0028), with the high (37.5 g/kg) or low (6.25 g/kg) doses ineffective. These effective ketamine or Yueju doses also reduced immobility time at 30 minutes after drug administration in independent groups (Figure 3(c), ANOVA, *F*_(2,24)_ = 4.875, *P* = 0.017, and *n* = 8–10). These doses of ketamine and Yueju, at 30 minutes after drug administration, did not change either locomotor activity (distance traveled) (Figure 3(d), AVOVA, *F*_(2,30)_ = 0.9903, *P* = 0.384, and *n* = 11) or time spent on the center part of open field (Figure 3(e), AVOVA, *F*_(2,30)_ = 0.0276, *P* = 0.973, and *n* = 11), a measurement of the level of anxiety. The open field behavior did not differ at 24 hours after drug administration (ANOVA, *F*_(2,21)_ = 0.915, *P* = 0.997, and *n* = 8). By 48 hours after drug administration, neither ketamine nor Yueju exhibited antidepressant response (ANOVA, *F*_(2,21)_ = 0.064, *P* = 0.938, and *n* = 8). 

### 3.4. BDNF Protein Expression Was Rapidly Increased by Yueju but Decreased at 24 Hours

**Figure 4:**
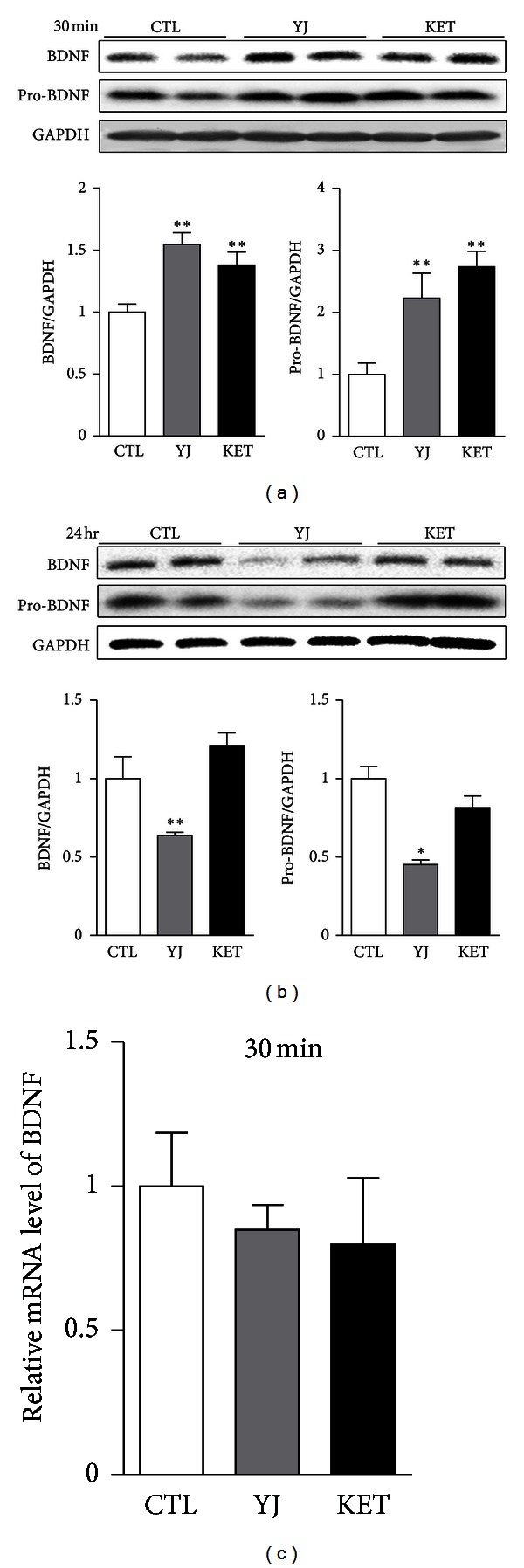
BDNF expression at 30 minutes and 24 hours after an acute administration of Yueju. (a) BDNF and BDNF precursor (pro-BDNF) protein expression levels in mouse hippocampus were determined by Western blot at 30 minutes after treatment with Yueju (YJ) or ketamine (KET). For BDNF, one-way ANOVA, *F*_(2,15)_ = 9.687; *P* = 0.002. For pro-BDNF, one-way ANOVA, *F*_(2,15)_ = 9.302; *P* = 0.002. (b) BDNF and pro-BDNF protein expression levels at 24 hours after drug treatment. For BDNF, one-way ANOVA, *F*_(2,15)_ = 18.81; *P* = 0.003. For pro-BDNF, one-way ANOVA, *F*_(2,15)_ = 9.416; *P* = 0.014. (c) BDNF mRNA expression was examined by quantitative RT-PCR in mouse hippocampus at 30 min after drug treatment. One-way ANOVA, *F*_(2,15)_ = 0.3193; *P* = 0.736. *P < 0.05; **P < 0.01, compared with the control group (CTL). Data represent mean ± SEM. *n* = 6.

To understand the molecular mechanism underlying rapid antidepressant effect of Yueju, we examined the expression of BDNF in the hippocampus at 30 minutes, 24 hours, and 48 hours after drug administrations. At 30 minutes (Figure 4(a)), there was a significant treatment effect on BDNF (ANOVA, *F*_(2,15)_ = 9.687, *P* = 0.002, and *n* = 6) or pro-BDNF expression (ANOVA, *F*_(2,15)_ = 9.302, and *P* = 0.002) in the hippocampus. Ketamine-treated mice showed significant increase in mature BDNF (*P* = 0.012) and pro-BDNF (*P* = 0.0002), and Yueju has the similar effects (*P* = 0.0008; *P* = 0.019, resp.). The rapid increase of BDNF in the hippocampus by Yueju may contribute to the rapid onset of antidepressant effect as shown in ketamine. However, at 24 hours (*n* = 6), the BDNF expression was decreased in Yueju-treated mice (*P* = 0.003), whereas BDNF expression in ketamine-treated mice did not differ from the vehicle mice (Figure 4(b)) (*P* > 0.05). BDNF level in Yueju-, ketamine- or vehicle-treated mice did not differ at 48 hours after drug administration (AVOVA, *F*_(2,15)_ = 0.2716; *P* = 0.764). In contrast to the increase in protein expression, the BDNF gene expression did not alter in either ketamine- or Yueju-treated mice at 30 min (Figure 4(c)) (*n* = 6, *F*_(2,15)_ = 0.3193, and *P* = 0.736). Taken together, it is likely that the increase in BDNF protein expression resulted from posttranscriptional regulation.

### 3.5. Yueju Rapidly Decreased eEF2 Phosphorylation but Remarkably Increased It at 24 Hours

**Figure 5:**
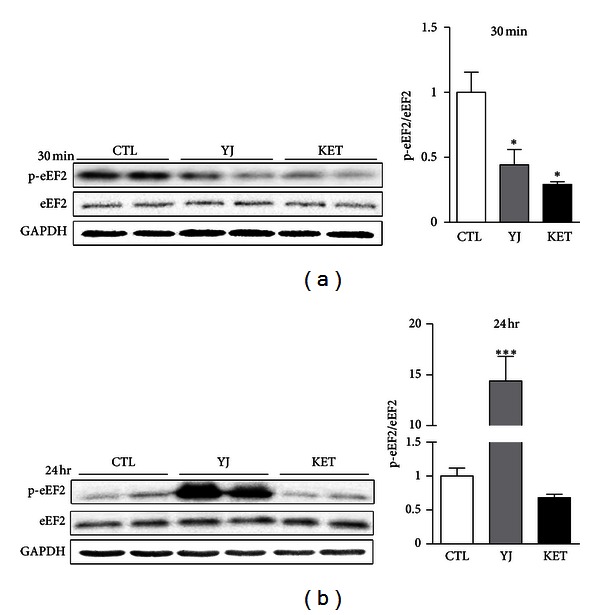
eEF2 phosphorylation at 30 minutes and 24 hours after an acute administration of Yueju. (a) Densitometric analysis of phosphorylated eEF2 (p-eEF2) normalized to total eEF2 in mouse hippocampus at 30 minutes after treatment with Yueju (YJ) or ketamine (KET). ANOVA, *F*_(2,15)_ = 10.95; *P* = 0.010. (b) Densitometric analysis of p-eEF2 normalized to total eEF2 in mouse hippocampus at 24 hours after drug treatment. ANOVA, *F*_(2,15)_ = 31.34; *P* = 0.001. *P < 0.05; ***P < 0.001, compared with the control group (CTL). Data represent mean ± SEM. *n* = 6.

Previous report suggests that ketamine deactivates eukaryotic elongation factor 2 (eEF2) kinase, leading to reduced eEF2 phosphorylation and desuppression of translation of BDNF. Here, we tested whether Yueju also regulated eEF2 phosphorylation in the hippocampus. The eEF2 phosphorylation was significantly influenced by the treatment at both time points of 30 minutes (AVOVA, *F*_(2,15)_ = 10.95, *P* = 0.010, and *n* = 6) and 24 hours (ANOVA, *F*_(2,15)_ = 31.34, *P* = 0.001, and *n* = 6) after drug treatment. At 30 minutes, similar to ketamine (*P* = 0.01), Yueju significantly reduced eEF2 phosphorylation (Figure 5(a), *P* = 0.045). In contrast, at 24 hours, eEF2 was substantially phosphorylated in Yueju-treated mice (*P* = 0.005), whereas the phosphorylated eEF2 still has a trend to decrease in ketamine-treated mice (Figure 5(b), *P* = 0.068). 

## 4. Discussion

Current conventional antidepressants act slowly, and it is urgent to develop the therapeutics that can quickly and enduringly treat depression. In the current study, we found that the ethanol extract of Yueju, an ancient antidepressant herbal medicine, alleviated the depressive symptoms in a rapid and lasting manner. We also identified the underlying molecular mechanisms. Yueju immediately increased BDNF protein expression in the hippocampus, which is likely associated with posttranscriptional regulation of BDNF synthesis by eEF2 phosphorylation.

The present study showed that Yueju is able to quickly and lastingly act as an antidepressant, using two animal behavioral paradigms of depression. We showed that one dose of Yueju significantly relieved the symptoms of depression in the learned helplessness paradigm, a depression model in which animals are exposed to unpredictable and uncontrollable stress, and subsequently develop coping deficits for aversive but escapable situations [18]. It represents a model with good similarity to the symptoms of depression, construct, and predictive validity. For conventional antidepressants, chronic and repeated administration is required for reduction of depressive symptoms in learned helplessness paradigm in animals [19]. In contrast, we found improvement of active avoidance escape responses and reducing freezing time by only one dose of Yueju, indicating a rapid antidepressant action of Yueju. TST is one of the most widely used acute models for quickly assessing the antidepressant effects [20]. In the test, the mouse is faced with a stressful, inescapable situation that has been suggested to engender a state of behavioral despair, akin to the hopelessness evident in depressives, and the immobility is interpreted as an episode of depression, or behavior despair. Despite lack of face and construct validity in modeling human depression, the TST behavior despair paradigm has great predictive validity for antidepressants. Conventional monoaminergic antidepressants decrease immobility acutely based on behavior despair paradigms. However, for conventional antidepressants, this effect does not last long, and it takes a long period time of repeated administrations before the antidepressant effects manifest in depressive patients or animals [20]. Here, we provided evidence that the antidepressant effect of Yueju as well as ketamine can sustain for 24 hours after a single administration of Yueju in ICR mouse strain. We did not observe the effect at 48 hours or 7 days after a single ministration of Yueju or ketamine in this strain (data not shown). It has been reported that Ketamine's lasting antidepressant effects are remarkably influenced by strains and dosages [9, 21]. Autry et al. [9] used forced swim test with effective doses of 3 mg/kg in C57Bl/6J mice, whereas another group reported that the 50 mg/kg is required for sustaining antidepressant effects for the same test in the same strain [22]. Réus et al. [23] used rats for the same test, with effective dose of 10 mg/kg, similar to Li et al. [8]. ICR mice or the related strain CD-1 mice have also been tested for ketamine previously, but only higher doses (30 mg/kg) have been reported to induce lasting antidepressant effect using tail suspension test paradigm [24]. We found that the dose of 50 mg/kg ketamine enabled the ICR mice to remain unanesthetized, and to sustain an antidepressant response. The possibility of a longer antidepressant effect of Yueju in other mouse strains cannot be ruled out. 

Yueju demonstrated the quick and lasting antidepressant effects similar to ketamine. As ketamine is the best characterized rapid and lasting antidepressant up to date, we used it as a positive control in the current study and investigated the molecular mechanisms underlying Yueju's quick and lasting antidepressant effects. It is now believed that the deficits in different forms of neuroplasticity, including synaptic mechanism and neurotrophic mechanism, are responsible for depression [25, 26], and thus neuroplasticity becomes the therapeutic target of antidepressants. BDNF is the best studied neurotrophic factor implicated in depression. Growing number of studies support that BDNF is necessary for mediating antidepressant effects [25, 27]. The rapid and enduring antidepressant effect of ketamine is also dependent on BDNF [9]. Our findings showed that, similar to ketamine, Yueju acutely increased the expression of BDNF at 30 minutes after administration. Conventional monoamine-based antidepressants also increase BDNF, but only after chronic drug administration [27, 28]. The fast induction of BDNF may underlie the fast action of Yueju. However, by 24 hours, the level of BDNF returned to normal by ketamine, whereas it was significantly reduced, compared to the control mice, by Yueju. The reduced BDNF in Yueju by 24 hours may be regulated by eEF2. Phosphorylation of eEF2 suppresses translation of proteins including BDNF. We found that Yueju rapidly downregulated the phosphorylation of eEF2. This dephosphorylation likely augments BDNF expression and thus exerts antidepressant effects [9]. By 24 hr, the eEF2 was remarkably activated in Yueju-treated mice, which led to suppression of translation, consistent with our observation of reduced expression of BDNF. However, Yueju- or ketamine-treated mice still show antidepressant response at this time. There was no change in open field test or cage activities in Yueju-treated mice at 24 hours with reduced hippocampal BDNF, which returned to normal level at 48 hours. It remained to know the other potential behavioral consequences from the temporary decrease of BDNF expression by Yueju. Taken together, the increase of BDNF expression is likely crucial for onset of antidepressant effects but may not be required for the sustaining antidepressant effects. 

 Previously, it has been reported that ethanol, but not aqueous, extract of Yueju has an antidepressant effect after a week-long administration [14]. Here, we provide further evidence that a single dose of the ethanol extract of Yueju has a quick and lasting antidepressant effect. This is in agreement with the clinical TCM practice in which Yueju is essentially administrated with pill made by mixture of the powdered herbs, instead of decoction, the most popular form of TCM herb medicine. In fact, many active components existing in herbs of Yueju are volatile oil that may be extracted with ethanol, such as *α*-cyperone and *β*-cyperone in Xiang Fu [29–31] and Atractylin and hinesol in Cang Zhu [32–34]. Some of them may be critically involved in the antidepressant action of Yueju. It is plausible that the effective components of Yueju act in concert to regulate glutamate and related neurotransmission and signaling, in a manner similar to ketamine. This surely warrants further investigations. 

 This is the first study demonstrating that a classic Chinese herb medicine is capable to exert a rapid and lasting antidepressant effect. We further showed that Yueju rapidly enhanced the expression of BDNF, indicating Yueju's upregulation of neuroplasticity. Although these findings are important for understanding therapeutic effects of Yueju on depression, the current study has several limitations. (1) We showed that Yueju was a fast acting and lasting antidepressant, using animal models of depression, but it is yet to determine the efficacy of Yueju on the chronic models of depression, which are believed to have better face and construct validity of clinical depression. (2) The intragastric dose used in mice in the current study is only about 1/3 of those published previously by other researchers [14]. Although sometimes the hypothetic equivalent or even higher intragastric dosages have been prescribed to patients, our dosage is still appreciably higher than the counterpart of recommended human dosage. Identification of the effective components of Yueju may lead to a reduced dosage to have a rapid antidepressant effects. (3) We identified the fast induction of BDNF expression as the key molecule by which Yueju exerts rapid antidepressant effects, but the upstream signaling as well as the contributions of other forms of neuroplasticity remain unknown. For example, mTOR signaling is also implicated in rapid and lasting antidepressant response [8]. It is yet to know if Yueju regulates mTOR signaling and related synaptic plasticity. (4) It remained to know how Yueju ethanol extract induces hippocampal BDNF expression changes, directly on neurons or glia, or indirectly via their metabolites. Future in vitro or in vivo studies should address the cellular mechanisms in more details. 

 In conclusion, we identified an ancient classic antidepressant herb medicine Yueju to act rapidly and lastingly in mice. To our knowledge, up to now, this is the only herb medicine demonstrating ketamine-like antidepressant effect. Moreover, Yueju quickly induces the expression of BDNF in the hippocampus, which may mediate the antidepressant effect in a fast manner. Yueju was invented eight hundred years ago and is still popularly prescribed in China, suggesting that Yueju is an effective and safe medicine in remedy of mood disorders including depression. Our findings lend a support for the efficacy of Yueju on quickly relieving depression symptoms and laid a foundation for further clarifying the underlying substrates. The present study also sheds a new light on the translational and clinic research on the quick and lasting antidepressant efficacy of Yueju.
